# Current Perspectives on Characteristics, Compositions, and Toxicological Effects of E-Cigarettes Containing Tobacco and Menthol/Mint Flavors

**DOI:** 10.3389/fphys.2020.613948

**Published:** 2020-11-19

**Authors:** Gurjot Kaur, Anshuman Gaurav, Thomas Lamb, Melanie Perkins, Thivanka Muthumalage, Irfan Rahman

**Affiliations:** ^1^School of Pharmaceutical Sciences, Shoolini University, Solan, India; ^2^Department of Environmental Medicine, University of Rochester Medical Center, Rochester, NY, United States

**Keywords:** e-cigarettes, menthol, mint, tobacco, toxicity

## Abstract

Electronic nicotine delivery systems/devices (ENDS) such as electronic cigarettes (e-cigarettes) have been made available globally, with the intent to reduce tobacco smoking. To make these products more appealing to young adults, many brands have added flavoring agents. However, these flavoring agents are shown to progressively result in lung toxicity when inhaled via e-cigarettes. While recent federal regulations have banned the sale of flavored e-cigarettes other than tobacco or menthol flavors, concerns have been raised about the health effects of even these flavors. In this review, we evaluate the current toxicological data with regard to effects upon exposure in animal models and *in vitro* cell culture for these popular flavorants. We have tabulated the current e-cigarette products containing these most common flavors (menthol, mint, and tobacco) in the market. We have also indicated the prevalence of tobacco and menthol-flavor use among e-cigarette users and highlighted the possible challenges and benefits that will result from new federal regulations.

## Introduction

E-cigarettes are a diverse group of products which allow for the inhalation of nicotine. Popular examples of these devices include cig-a-likes, vape pens, and mods. In addition to nicotine, e-cigarette aerosols contain many other chemicals. These include, but are not limited to, flavors, humectants, such as propylene glycol, formaldehyde, acrolein, and specific nitrosamines.

These devices can deliver various concentrations of nicotine, dependent on the various constituents of the e-cigarette (Kaur et al., 2018). As of January 2014, there were 466 unique brands of electronic nicotine products and this number increased, on average, by 10.5 brands per month from August 2013 to January 2014 (Barrington-Trimis et al., 2014). There is an extremely diverse range of e-cigarette flavors available in the US market; with over 8,000 flavors available from mint to fruit to dessert flavors, brands have established a broad appeal to both adults and children (Kaur et al., 2018). Although adolescents have been made aware of the risks of e-cigarettes, many continue to hold relatively favorable attitudes toward e-cigarettes (Gorukanti et al., 2017). According to the Health Information National Trends Survey (HINTS) data reported in 2015, Americans who believed that e-cigarettes were less addictive than tobacco cigarettes were almost 2.5 times more likely to try e-cigarettes than those who believed e-cigarettes were equally or more addictive than tobacco cigarettes (Lewis-Thames et al., 2020). In addition, e-cigarette users often assume that it is more acceptable to use e-cigarettes both indoors and outdoors in contrast to conventional cigarettes that can be used only outdoors. Common misconceptions among adolescents also include the belief that e-cigarettes are safer than conventional cigarettes, that they help people quit smoking, and that they contain little or no nicotine (Gorukanti et al., 2017).

Of particular concern is the widespread use of e-cigarettes among high school and middle school students. The 2011–2018 National Youth Tobacco Survey (NYTS) showed an increase in e-cigarette use in both high school and middle school students, 20.8 and 4.9%, respectively. Specifically, between 2017 and 2018, there was a 78 and 48% increase in e-cigarette use by high school students and middle school students, respectively (Cullen et al., 2018).

Young e-cigarette users may be influenced into adopting this harmful habit by the marketing of e-cigarette manufacturing companies. These companies often use harmful marketing strategies to increase sales, i.e., displaying e-cigarettes as safer alternatives to other forms of smoking while also promoting appealing flavors (Bhalerao et al., 2019). According to the NYTS held jointly by the U.S. Food and Drug Administration (FDA) and the Centers for Disease Control and Prevention, around 3.6 million students were using e-cigarettes in 2018 (Bhalerao et al., 2019). In the 2019 NYTS, e-cigarette usage in high school students reportedly increased to 27.5% and in middle school students to 10.5%. Approximately 59% of high school students and 54% of middle school students used JUUL as their usual device, with both groups preferring fruit flavors (Cullen et al., 2019).

Due to this increase in e-cigarette usage among adolescents and the high preference for flavored e-cigarettes, the FDA took action to limit access to these devices. In January 2020 the administration ruled that the sale of any flavored, cartridge-based electronic nicotine systems (ENDS), other than tobacco and menthol flavors, would be prohibited (Enforcement Priorities for Electronic Nicotine Delivery System (ENDS) and Other Deemed Products on the Market Without Premarket Authorization). Due to the ban on flavored, cartridge-based ENDS, concern has now largely shifted to the currently available flavors, menthol and tobacco.

## Menthol and Tobacco Flavor Usage

E-cigarettes that contained 3.5% menthol have been shown to have a greater likelihood of Usage compared to e-cigarettes without menthol. Menthol usage (0.5–3.5%) resulted in a significant improvement in taste and thus, higher nicotine concentrations (12 mg/ml) could be used (Krishnan-Sarin et al., 2017). Interestingly, unit sales of menthol e-cigarettes as a percent of all units sold remained stable from 2012 (39.9%) to 2016 (36.6%) (Kuiper et al., 2018). But first flavor purchases have altered over time. Tobacco and menthol flavors have been the highest and second-highest purchased flavors approximately 5 years ago. Fruit flavors ranked as the top choice for the last 3 years and even more prominently in the last year. Tobacco and menthol preference has decreased over time, with menthol ranked fourth and tobacco as the second (Russell et al., 2018). Among adults, the most common flavor used within the past 30 days of the survey was menthol, while in youth, menthol was the fourth most common flavor (Schneller et al., 2018). Currently, there is great diversity in the e-cigarette flavors within menthol and tobacco categories.

To evaluate the current market share and usage of the most common flavors, i.e., tobacco, menthol and mint, we performed a market investigation of brands that sell ENDS products with these three flavors (Supplementary Table 1). As stated, there is a considerable portion of marketed ENDS products that have either of the three flavors. Out of the three flavors, tobacco flavoring captured the most ENDS products, leading in e-liquid, e-liquid with salts, pods, and cartridges categories. It may be assumed that this preference and thus availability is due to public preference for tobacco flavor. This may arise from the desire to replace the sensation of tobacco in the absence of conventional cigarettes. Nevertheless, menthol and mint are also common flavors and closely followed tobacco in various categories.

## Current Safety Status of the Most Common Flavors in E-Cigarettes

In e-liquids that had at least one flavoring chemical with a concentration greater than 10 mg/ml, menthol was present in 50% of the samples. Menthol concentration has been shown to be cytotoxic in 34% of refill fluids (Omaiye et al., 2019). In addition, mint and menthol ENDS are shown to contain pulegone. However, the FDA has already banned synthetic pulegone as a food additive as it is a known carcinogen (Jabba and Jordt, 2019). In traditional cigarettes, studies have shown that menthol increased the reinforcing nature of nicotine on smoking behavior (Ahijevych and Garrett, 2010). Along with this reinforced nature, menthol in traditional cigarettes can result in an increase in nicotine dependence compared to non-menthol cigarette smokers (Villanti et al., 2017). Menthol cigarette smoking was found to be most prevalent in adolescent smokers between 2008 and 2010. There was generally a more rapid decline in non-mentholated cigarette smoking than in mentholated cigarette smoking (Giovino et al., 2015). A similar scenario is expected in menthol containing e-cigarettes.

**a. Tobacco Flavors**

As with many other e-cigarette flavors, tobacco flavors are often marketing with enticing names such as King Pin, Havana Cigar, Classic Tobacco, Renegade, Wizard’s Leaf, and Cowboy. An extensive list of various brands’ tobacco flavors in given in Supplementary Table 1.

Several studies have sought to investigate the cellular toxicity of e-cigarette tobacco flavors. Some have found that epithelial cells exposed to tobacco flavor vapor showed increased levels of cell death over a period of several hours to several weeks (Yu et al., 2016). Others have shown that exposure to the tobacco flavoring can cause inflammatory responses in cells such as fibroblasts (Sundar et al., 2016; Table 1). The general results reported among available studies looking at tobacco flavors often included decreased cell viability, decreased numbers of cells, and increased inflammation after exposure (Table 2).

**TABLE 1:** Current literature on mouse inhalation toxicology after flavorant exposure.

**References**	**E-Cigarettes types**	**Flavoring agent**	**Mouse inhalation toxicology studies**
Lerner et al., 2015	Aerosol	Tobacco and menthol (concentration not mentioned) and other flavors.	8 weeks old C57BL/6J mice, whole-body inhalation exposure to e-cigarette aerosol (16 mg nicotine), 5 h/day for 3 successive days. For control, mice were exposed to air. **Finding:** flavoring contributed to enhanced OX/ROS reactivity in mice.
Zelikoff et al., 2018	Aerosol	Tobacco flavor (concentration not mentioned)	8–9 weeks old pregnant C57BL/6 mice, whole-body inhalation exposure to aerosol (without or with nicotine 13 mg/ml). Mice were exposed for 3 h/day, 5 days/week from pregnant to gestation (about 3 weeks). For control, mice were exposed to filtered air. **Finding:** Disruptions in the development of CNS may be attributed to presence of flavorings however no experimental evidence is provided in the publication.
Chen et al., 2018	Aerosol	Tobacco flavor (concentration not mentioned)	Female Balb/c mice were exposed to e-vapor (without or with nicotine 18 mg/ml) twice daily for 6 weeks prior to mating until pups weaned. For control, mice were exposed to room air. **Finding:** Some part of increased IL-1β, IL-6 and TNF-α release in mother’s lung could be attributed to either humectant or flavoring agent although direct experimental evidence for role of flavorant was missing.
Glynos et al., 2018	Aerosol	Tobacco blend flavor (4%)	Eight-to-twelve- week-old male C57BL/6 were exposed to e-cigarette aerosol (base with nicotine 18 mg/ml) 4 times a day with 30-min smoke-free intervals for 3 days or 4 weeks. For control mice were exposed to air. **Finding:** Change in nicotine induced Bronchoalveolar lavage fluid cellularity, Muc5ac production, lung oxidative stress markers get exacerbated due to presence of tobacco flavor in test samples.

*The table lists currently available toxicology studies in presence of flavors (menthol, tobacco or mint). The concentration of flavoring agent is also mentioned.*

**TABLE 2:** Current literature on *in vitro* inhalation toxicology after flavorant exposure.

**References**	**E-Cigarette types**	**Flavoring agent**	**Human *in vitro* toxicology studies**
Willershausen et al., 2014	E-Liquid	Menthol (10 μg/ml), Hazelnut, Lime flavors	Human periodontal ligament fibroblasts (HPdLF) were incubated up to 96 h with the different liquids (base with nicotine concentration-10μg/ml). For control, fibroblasts were treated with PBS. Cell viability was measured. **Finding:** In cell visualization test, ATP was reduced in fibroblasts due to presence of menthol flavor.
Lerner et al., 2015	Aerosol	Tobacco and menthol (conc. not mentioned) and other flavors.	Human bronchial airway epithelial cells (H292) and human fetal lung fibroblasts (HFL1) treated with various flavored e-liquids for 24 h and examined for morphological changes/cell stress. **Finding:** Reduction in cell number and increase in cell size and vacuolarization observed in e-liquid treated cells. Presence of cinnamon flavoring agent increased IL-8 levels but not tobacco or other flavors in HFL-1.
Yu et al., 2016	Aerosol	Classic tobacco, red American tobacco flavors (concentration not measured in the study)	Normal epithelial cells (HaCaT) and head and neck squamous carcinoma cell line (UMSCC10B, HN30) treated with nicotine free and nicotine containing e-cigarette vapor (base with nicotine concentration 0–12 mg/ml) from 48 h to 8 weeks. Cytotoxicity and Genotoxicity was assessed. **Finding:** Regardless of e-cig vapor nicotine content, cells viability was reduced along with increased necrosis and apoptosis due to presence of substituents and tobacco flavors in test samples.
Leigh et al., 2016	Aerosol	Tobacco, Menthol, (concentration not measured in the study) and other flavors - Pina colada, Coffee and Strawberry	H292 human bronchial epithelial cells exposed to 55 puffs ENDS (base with nicotine content 24 mg/ml). For control, cells were exposed to air using air-liquid interface system. Cell viability, metabolic activity and inflammatory mediators were assessed. **Finding:** All flavors significantly caused toxicity (increased inflammatory mediators, reduced cell viability and metabolic activity). Strawberry flavored e-cigarette vapors were most cytotoxic.
Sundar et al., 2016	Aerosol	Classic tobacco, Magnificent menthol flavors (concentration not measured in the study)	Human periodontal ligament fibroblasts and human gingival epithelium progenitors pooled exposed to aerosol (nicotine content in classic tobacco and magnificent menthol were ‘16’ mg and ‘0’mg). For control cells were exposed to air. Oxyblots was used to determine protein carbonylation. IL-8 and PGE2 were determined by ELISA. **Finding:** Inflammatory and prosenescence responses were increased due to the presence of classic tobacco and magnificent menthol flavor in test samples.
Bengalli et al., 2017	Aerosol	Tobacco, Mint, and Cinnamon flavors (concentration of Menthol 5–10%, Cinnamon 1.5%)	Cultured human lung adenocarcinoma cells A549 and NCI-H441 exposed to e-cig vapor (base with nicotine 0–18 mg). MTT assay and Alamar Blue tests were performed to analyse cell viability. Pro-inflammatory cytokines release and alveolar-blood barrier integrity were assessed. **Finding:** Nicotine itself had almost no influence on toxicity but flavors were responsible for modulation of toxicity response.
Leslie et al., 2017	Aerosol	Mint, Menthol and other flavors- Cherry, Crisp mint, Vanilla, Apple, Strawberry flavors (concentration not measured in the study)	Human-derived bronchial epithelial cell lines, BEAS-2B, IB3-1, C38 and CALU-3 and human derived fibroblast cell Line-Wi-38, exposed to vapor extract of e liquid (base with nicotine content: 0.8–16 mg/ml) for 24 h. Viability was assessed by using a standard XTT assay. **Finding:** Cytotoxicity induced due to presence of tobacco, cherry and strawberry flavor in both test and control group. 100% strawberry flavored e-cigarette exposure proved to be more cytotoxic in both test as well as control samples.
Rowell et al., 2017	Aerosol	Vanilla tobacco, Menthol tobacco variant, Solid menthol and other flavors-Captain black cigar, Peanut Butter cookie, T-bone, Popcorn, Black licorice, Energon, Banana pudding, Kola, Hot cinnamon candies (concentration not measured in the study)	Lung epithelial cell line (CALU3) exposed to 13 different flavored e-liquids (base with nicotine content 12 mg/ml). Cell proliferation/viability tested using MTT assay. Measurement were recorded after 24 h. **Finding:** menthol tobacco and flavors-Banana pudding (southern style), kola and hot cinnamon candies flavors proved to have negative effect on cell proliferation and cell viability in test samples. After 24 h of exposure, menthol tobacco and hot cinnamon candies flavors showed cytotoxicity in confluent CALU3 cultures.
Miyashita et al., 2018	Aerosol	Tobacco flavors (concentration not measured in the study)	Alveolar type II epithelial cell line (A549) and bronchial epithelial cell line (BEAS-2B) were exposed to e cig vapor (nicotine- 24 mg/ml). Lactate dehydrogenase release was measured to assess cell membrane integrity.
			**Finding:** Regardless of nicotine, nicotine free e–cigarette increased PAFR-mediated pneumococcal adhesion to epithelial airway cells, possibly, due to presence of other chemicals and/or tobacco flavor in test samples.
Behar et al., 2018	Aerosol	Tobacco, Mint and other flavors-Chocolate, Vanilla, Caramel, Coffee etc. (concentration not measured in the study)	Human pulmonary fibroblasts, lung epithelial cells (A549) and human embryonic stem cells were used in this *in vitro* study. Cells were exposed to e-cig vapor (base with nicotine content 6–24 mg/ml). Cytotoxicity measured using the MTT assay. **Finding:** Cytotoxicity induced due to presence of tobacco, mint and other flavors in test samples.
Fetterman et al., 2018	Aerosol	Menthol and other flavors: Vanilla, Cinnamon, Strawberry Butter, Banana, Spicy, burnt (concentration not measured in the study)	Endothelial cells were exposed to aerosol. For controls vehicles were matched to flavoring. Cell death, ROS production, expression of the pro-inflammatory interleukin-6, and nitric oxide production were measured. **Finding:** Menthol flavored tobacco cigarettes stimulated nitric oxide production. Endothelial cell dysfunction was induced due to presence of flavors (vanillin, menthol, cinnamaldehyde, clove, and burnt) in tobacco products.
Muthumalage et al., 2017	E-liquid	Tobacco, menthol and other flavors- Alcohol, Berry, Cake, Candy, Coffee/Tea, Fruit flavors (concentration not measured in the study)	Monocytic cells from human pleural tissue (U937) and human monocyte macrophage cell line Monomac-6 (MM6) treated with e-liquid. Cell viability, free ROS and inflammatory cytokines were measured. **Finding:** Cell-free ROS level were elevated due to presence of flavoring chemicals (e.g., tobacco) in test samples. Mixing of e-liquid flavoring chemicals leads to more cytotoxicity as compared to unique flavor in test samples. Exposure to flavored e-liquid without nicotine induced cytotoxicity and cytokine release in cells for flavors other than tobacco.
Otero et al., 2019	E-liquid	Menthol and other flavors- Mango, Watermelon, Cinnamon, Apple, Coffee (concentration not measured in the study)	Human MG-63 and Saos-2 osteoblast-like cells were treated with e-liquid (nicotine content 24 mg/ml) for 48 h. Key osteoblast markers, RUNX2 and Col1al, changes in cell viability were assessed. **Finding:** Cell viability is reduced with all flavors containing e-liquids. mRNA expression was upregulated due to coffee-flavored and fruit-flavored e-liquids in cells. Collagen type I protein was more expressed on exposure to fruit-flavored Mango Blast e-liquid. Cinnamon-flavored were the most toxic.
Zahedi et al., 2019	E-liquid/Aerosol	Menthol and Tobacco flavors (concentration 1%)	Neural stem cells exposed to e-liquid/aerosol (e-liquids had 44 mg/mL nicotine, whereas aerosols had nicotine of 110 μg/mL). Mitochondrial superoxide levels, mitochondrial protein oxidation, mitochondrial membrane potential, mitochondrial nucleoids and mtDNA damage were measured. **Finding:** An increase in lysosome co-localization, decrease in degradation and increased autophagic load due to flavors.
Al-Saleh et al., 2020	E-liquid	Menthol (0.004-4769.326 μg/g) in Tobacco and other flavors- Vapes Lab Sweet Tobacco Sour Straws, Honey Crème, Vanilla Custard, Coffee crème, Banana ice, Turkish, Craze shake, Double apple, Gemini, Fruitz, HYDRA, Chai Karak, Rainbow grape, Irish, Pineapple, chocolate, Bedrock, Kiberry Yogurt, Milk & Strawberry Wonder, and derived flavors	Human lymphoblastoid TK6 and Chinese hamster ovary cells treated with a total of 68 e-liquid representing 33 brands with nicotine content up to 8 mg. Menthol concentrations were measured in all flavor variants. PAEs, DL-menthol, nicotine, DNA damage, chromosome breakage, and cell viability were assessed. **Finding:** In TK cells out of 63 flavors, 47 flavors induced with DNA damage and 26 flavor reduced cell viability. Even at low levels, menthol was found to be associated with increased DNA damage and reduced cell viability.
Go et al., 2020	E-liquid	Tobacco, menthol flavors (concentration not measured in the study although e-liquid tested concentrations are listed)	HMEECs (human middle ear epithelial cells) exposed to flavored e-liquid for 24 hours at various concentrations (0.01 to 10%). Control group were not exposed to e-liquid. **Finding:** Reduced cell viability with increasing tobacco or menthol flavored e-liquid concentrations. mRNA levels of genes encoding epithelial sodium channels in HMEECs were decreased due to both flavored e-liquid exposure. In comparison to menthol-flavored e-liquids, tobacco flavored e-liquid increased the levels of
			autophagosome marker followed by cell death. Tobacco flavored e-liquid increased the level of inflammatory cytokine and mucin production. Flavored e-liquid induced apoptosis and autophagy reactions.
Lamb et al., 2020	Pods	Menthol, Tobacco (individual chemical concentrations were measured in the study)	Beas2b cells (lung epithelial cells) exposed to e-cigarette pods with nicotine concentration of 5% for 22 minutes. For control cells were exposed to air. Puff volume was 55 ml/min. Cells were kept for 8 min to gain exposure of 30 min. **Finding:** Menthol flavored pods induced mitochondrial dysfunction, reduced respiration in mitochondria, reduced OXPHOS in Beas2b cells. Tobacco pods exposure did not cause any alternation in energetics of mitochondria.

*The table lists currently available toxicology studies in presence of flavors (menthol, tobacco or mint). The concentration of flavoring agent, when available, is also mentioned.*

**b. Menthol/mint Flavors**

Menthol and mint flavors are likewise marketed to appeal to both adolescents and adults. Examples of brand-specific flavor names in this category include Arctic Blast, Mountain Chill, Polar Bear, Kringle’s Curse, Blue Slushie Iced, and Candy Cane. Additional examples of mint and menthol flavors are listed in Supplementary Table 1.

The chemical menthol is often used to impart a mint flavor and may be used in combination with other flavoring chemicals. One study investigating the cellular effects of exposure to menthol flavors found that lung epithelial cells exposed to a pod menthol flavor showed decreased mitochondria function with resulting decreased respiration in the mitochondria (Lamb et al., 2020). Another showed that exposure to these flavors resulted in a decrease in the amount of ATP in a sample of fibroblasts (Willershausen et al., 2014). Another study observed increased inflammation in bronchial epithelial cells after exposure to menthol (Leigh et al., 2016). Most studies utilizing menthol and mint flavors showed general trends such as higher levels of DNA damage and increased cell death following exposure (Table 2).

**c. E-cigarette or Vaping Product Use-Associated Lung Injury (EVALI) chemicals and toxicity based on forensic chemistry and biology**

**FIGURE 1:**
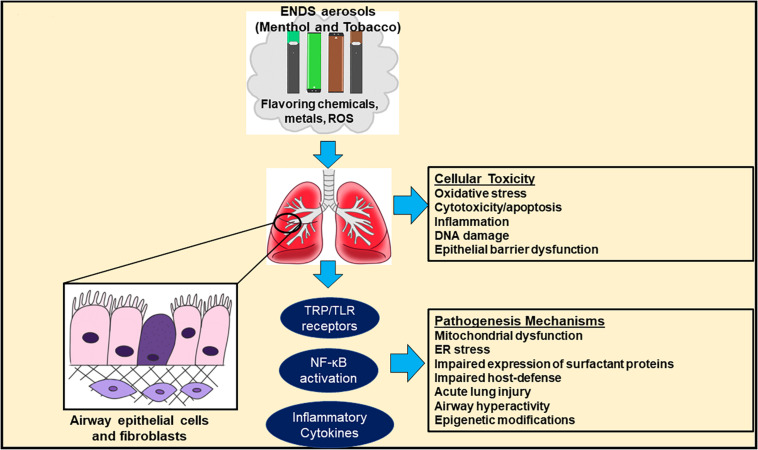
Cellular mechanisms of toxicity for menthol and tobacco flavors. The figure summarizes the current cellular pathways and pathogenesis mechanisms involved in cellular toxicity for menthol and tobacco flavorings.

**FIGURE 2:**
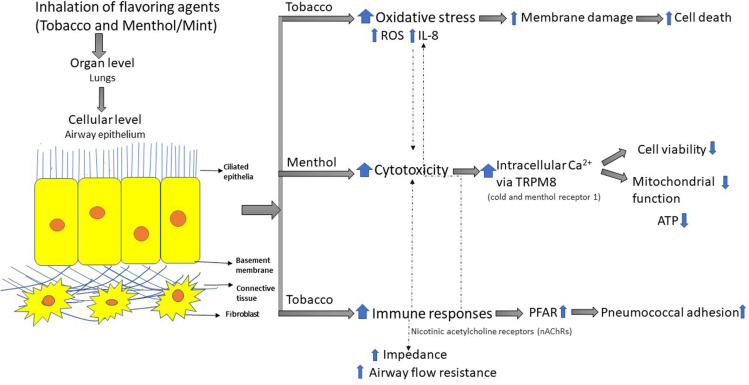
Cellular dysfunction by inhaled flavoring agents. Flavoring chemicals induced oxidative stress and inflammatory responses are associated with immune-responses via alterations in barrier tight junction dysfunction in the lung. Mitochondrial dysfunction and other cellular alterations can lead to susceptibility to infections. Tobacco flavor (nicotine) activate Nicotinic acetylcholine receptors (nAChRs) and menthol activate cold and menthol receptor 1 (i.e. via TRPM8) leading to various downstream cellular signaling events.

Additional chemicals have been implicated in the e-cigarette or vaping product use associated lung injury, or EVALI, observed in some e-cigarette users. Some of these include vitamin E acetate, THC, various hydrocarbons, terpenes, pesticides, plasticizers, and assorted metals (Muthumalage et al., 2020a). Among these components, vitamin E acetate has become a popular subject of research. This chemical especially is under scrutiny for its role in lung injury as it has been observed in sampled patients. Studies into the toxic effects of inhaled acetate have shown that this chemical may play a role in inducing inflammation responses in cells (Muthumalage et al., 2020b). Other vaping chemicals, such as medium-chain triglycerides, have also been the subject of study in relation to their possible role in this severe form of lung injury. One study investigated the effects of both this group of chemicals and vitamin E acetate on human cells. The exposure of pulmonary epithelial cells and immune cells to these chemicals resulted in harmful effects to the cells via lipid mediators. Decreased barrier function among the epithelial cells was observed, as well as a general increased immune response via activation of Toll-like receptor (TLR) or transient receptor potential (TRP)-like channels (Muthumalage et al., 2020b; Figures 1, 2).

### Toxicological Evaluation of ENDS

To assess the toxicology associated with the usage of flavors added to e-cigarettes, we compiled and exhaustively analyzed original research articles associated with the topic. We divided the original data into animal exposure studies (Table 1) and *in vitro* cell culture studies (Table 2).

#### Current Status on Flavor Induced Toxicology in Mice

We could identify four original articles that observed flavor (tobacco, menthol, or mint) associated toxicity in mice (Table 1). Lerner et al. (2015) tested six tobacco flavored e-liquids and one menthol flavored e-liquid in mice through whole-body inhalation exposure and observed increased OX/ROS reactivity along with oxidative stress in the presence of flavoring agents in the absence of nicotine. Zelikoff et al. (2018) demonstrated a sans nicotine effect on the developing central nervous system (CNS) in C57BL/6 mice and hypothesized the possible role of flavoring agents in the stunted CNS growth. Although the direct experimental evidence for the involvement of flavoring agents could not be provided by the other two studies (Chen et al., 2018; Glynos et al., 2018), it was shown that the presence of flavoring agents might have a role in increased IL-1β, IL-6, and TNF-α levels and other oxidative stress markers in lungs. We did find many previous studies that used flavored e-liquids to assess the effect of nicotine as part of ENDS product in mice; however, these earlier studies only compared nicotine treatments and generally, did not mention the exact concentrations or composition of flavoring agents (Table 3). Interestingly, recent studies also lack in the measurement of flavorants (Table 1). In general, the use of a 1–5% tobacco flavor did not yield any marked differences in measurable outcomes of testis toxicity (El Golli et al., 2016b; Rahali et al., 2018) or hepatic function (El Golli et al., 2016a). Similar results were obtained in DNA damage and mitochondrial dysfunction with tobacco flavor (concentration not mentioned) (Espinoza-Derout et al., 2019), and pro-senescence phenotypes (Sundar et al., 2016; Jabba and Jordt, 2019; Lucas et al., 2020) leading to transformational changes of normal cells by e-cig derived mint/menthol flavor toxicants.

**TABLE 3:** Current literature on flavor induced inhalation toxicology.

**References**	**E-cigarettes types**	**Flavoring agent**	**Toxicology studies**
**Mice studies**			
Sussan et al., 2015	Aerosol	Menthol flavor (concentration not measured in the study)	8-week-old C57BL/6 mice whole-body inhalation exposure to aerosol (1.8% nicotine), 1.5 h every time, twice/day for 2 weeks. For control mice were exposed to filtered air. **Finding:** No change in nicotine induced oxidative stress and moderate macrophage-mediated inflammation due to the presence of menthol flavor in test samples.
El Golli et al., 2016b	E-liquid	1–5% Tobacco flavor	Male Wistar rats (160 ± 20 g), Intraperitoneal injection e-liquid (without or with nicotine 18 mg/ml), 0.5 mg/kg of body weight, once/day for 4 weeks. For control mice were treated with physiological saline (500 ml) intraperitonially. **Finding:** No change in nicotine induced testis toxicity due to presence of tobacco flavor in test samples.
El Golli et al., 2016a	E-liquid	1–5% Tobacco flavor	Adult Wistar rats (160 ± 20 g), Intraperitoneal injection e-liquid (without or with nicotine- 18 mg/ml), 0.5 mg/kg of body weight, once/day for 4 weeks. For control mice were treated with physiological saline (500 ml) intraperitonially. **Finding:** No change in nicotine induced hepatic function due to presence of tobacco flavor in test samples.
Lauterstein et al., 2016	Aerosol	Classic tobacco flavor (concentration not measured in the study)	9-weeks-old pregnant C57BL/6 mice exposed to e-cig aerosol (without or with nicotine 13–16 mg/mL), 3 h/day, 5 days/week from pregnant to gestation (about 3 weeks), continued exposure from postnatal days to lactation. For control mice were exposed to filtered air. **Finding:** No change in nicotine induced chronic neuropathology and sex dependent gene expression due to presence of tobacco flavor in test samples.
Larcombe et al., 2017	Aerosol	Tobacco flavor (concentration not measured in the study)	Between the ages of 4 and 12 weeks, female BALB/c mice were exposed to one of four e-cigarette aerosols (nicotine 12 mg/ml). Mice were exposed for 1 h/day, 5 days/week up to week 10 of life. From week 11 to 12 of life exposures were increased to 1 h, twice daily, 5 days/week. Twelve mice were exposed to each exposure regime. For control mice were exposed to medical air. **Finding:** - No change in nicotine induced decrease in parenchymal lung function at both functional residual capacity and high transpiratory pressures due to presence of tobacco flavor in test samples.
Rau et al., 2017	Aerosol	Classic tobacco flavor (concentration not measured in the study)	6-week-old male (180–200 g) Sprague Dawley rats exposed to e cig vapor (nicotine content 12 mg/ml medium exposure and 24 mg/ml high exposure) for 4 weeks. At 5th week, flap survival was evaluated. For control mice were exposed to room air. **Finding:** No changes in nicotine induced necrosis in dorsal flaps due to presence of tobacco flavor in test samples.
Rahali et al., 2018	E-liquid	1–5 % Tobacco flavor	Male Wistar rats (160 ± 20 g), Intraperitoneal injection e-liquid (nicotine concentration-18 mg/ml) for 4 weeks. For Control rats were given i.p. injection of NaCl in a 9 g/l concentration. **Finding:** No change in nicotine induced testis toxicity due to presence of tobacco flavor in test samples.
Qasim et al., 2018	Aerosol	Menthol flavor (concentration not measured in the study)	C57BL/6 10J male mice (10 weeks old) exposed to e cig vapor (nicotine concentration- 18 mg/ml) over 2 sessions, i.e., 200 puffs per day, and lasted for 5 days/1 week. For control mice were exposed to clean air. **Finding:** No change in nicotine induced platelets hyper activation, activation of the aIIbb3 integrin, shortened thrombosis occlusion and bleeding times due to presence of menthol flavor in test samples.
Espinoza-Derout et al., 2019	Aerosol	Tobacco flavor (concentration not measured in the study)	Apo lipoprotein E knockout (ApoE-/-) mice were exposed to e cig aerosol (without nicotine or with 2.4% nicotine) for 12 weeks. DNA damage and mitochondrial dysfunction were assessed. For control mice were exposed to saline aerosol. **Finding:** No change in nicotine induced oxidative stress in liver cells, mitochondrial DNA mutation, reduction in cellular organelles and mitochondrial vacuolization in hepatic cells due to presence of tobacco flavor in test samples.
Nguyen et al., 2019	Aerosol	Tobacco flavor (concentration not measured in the study)	24 female Balb/C mice (7 weeks old) and animals were divided into three treatment group and exposed to e-cigarette aerosols (18 mg nicotine). At 12 weeks old offspring’s behavioral assessments were performed. From offspring at P1 (birth), P20 (weaning), and Week 13 brain tissue and plasma were collected. For control mice were exposed to air. **Finding:** No change in nicotine induced reduction in neuronal cell numbers of the dorsal hippocampus (cornu ammonis 1 region) and reduction in global DNA methylation due to presence of tobacco flavor in test samples.
Li et al., 2019	Aerosol	Tobacco flavor (concentration not measured in the study)	Female Balb/C mice (7 weeks old) were exposed to e-cig vapor generated from e-liquid (nicotine concentration- 18 mg/ml) for 6 weeks prior to mating until pups weaned. For control mice were exposed to room air. **Finding:** No change in nicotine induced oxidative stress, inflammation and fibrosis in adult offspring due to presence of tobacco flavor in test samples.
Ramirez et al., 2020	Aerosol	Menthol flavor (concentration not measured in the study)	10 to 12 weeks old C57BL/6J mice were exposed aerosol of e-cigarette pods (concentration of 5% by weight). Mice were exposed to 70 puffs daily for 2 weeks with 3 s puff duration and 25 s of interval time. For control mice were exposed to clean air. **Finding:** No change in nicotine induced platelet secretion, integrin GPIIb/IIIa activation and phosphatidylserine expression due to presence of menthol flavor in test samples.
**Human *in vitro* cells**			
Wu et al., 2014	E-liquid	Tobacco flavor (concentration not measured in the study but tested e-liquid concentrations are given)	Human tracheobronchial epithelial cells isolated from bronchi and trachea. Tracheas and bronchi were digested with ice-cold DMEM (0.2% protease). Cells were treated with e-liquid (without nicotine or with nicotine 18 mg/ml) for 48 h. Toxicity was assessed by measuring lactate dehydrogenase (LDH) levels and IL-6 protein levels by ELISA. For control cells were infected with HRV-16 at PBS (control) for 24 h. **Finding:** No change in nicotine induced IL-6 release in human airway epithelial cells and suppressed expression of SPLUNC1 due to presence of tobacco flavor in test samples.
Putzhammer et al., 2016	Aerosol	Tobacco, Menthol flavors (concentration not measured in the study)	Human umbilical vein endothelial cells (HUVECs) exposed to hydrophilic fraction of e-cigarette vapor (base with nicotine content 6–24 mg/ml). Cell death induction, occurrence of intracellular reactive oxygen species, proliferation rates, and cell morphology were analyzed. **Finding:** No change in nicotine and base induced alternations in cell morphology, inhibition of cell proliferation, induction of oxidative stress due to presence of tobacco and menthol flavor in test samples.
Lerner et al., 2016	Aerosol	Tobacco flavor (concentration not measured in the study)	E-cig aerosols (nicotine content 16 mg/ml) containing copper nanoparticles exposed to human lung fibroblasts (HFL-1) using an air-liquid interface culture system. For control fibroblasts were exposed to air. **Finding:** No change in nicotine induced nuclear DNA fragmentation and inflammatory cytokines IL-8, IL-6 release due to presence of tobacco flavor in test samples.
**Human reports**			
Vardavas et al., 2012	Cartridge	1–5 % Tobacco flavor	This was laboratory based experimental vs. control group study. Thirty participants participated in this study (experimental group, *n* = 30) (control group, *n* = 10). Experimental group were asked to use e-cig cartridges (less than 10% nicotine) for 5 min. For control, users were asked to use e cigarette cartridge without vapor. **Finding:** Change in nicotine induced total respiratory impedance, overall peripheral airway resistance due to presence of tobacco flavor in test samples was not part of experimental design.
Moheimani et al., 2017	Aerosol	Tobacco, strawberry flavor (concentration not measured in the study)	Total of 39 healthy non-smoker participants between the ages of 21 and 45 years. Fifteen subjects used the Green smoke cigalike device with tobacco flavored liquid with 1.2% nicotine. Eighteen subjects used a more efficient second-generation pen like device with strawberry flavoring with 1.2% nicotine. For control, users were asked for puffing without e-liquid. **Finding:** No change in nicotine induced altered cardiac sympathovagal balance toward sympathetic predominance due to presence of tobacco and strawberry flavor in test samples. However, flavorant controls were not part of the study.
Kerr et al., 2019	E-liquid	Tobacco flavor (concentration measured through GC/MS in the study)	A cross over study between tobacco cigarette users and e-cigarette users. Twenty healthy male smokers (before and after e-cig usage) were exposed to e-liquid (base with average nicotine concentration 17.27 mg/ml). Blood pressure, heart rate microvascular reactivity, reactive hyperaemia index, augmentation index and respiratory functions were assessed. **Finding:** Flavorant based effects were not tested. No change could be shown in nicotine induced micro particle formation indicating endothelial injury and altered peak expiratory flow due to presence of tobacco flavor in test samples.

*The table lists currently available toxicology studies wherein flavors (menthol, tobacco or mint) were used although the experimental design did not include observations for presence of flavoring agents. The concentration of flavoring agent, when reported, is also mentioned.*

Additional studies into the effects of e-cigarette exposure with developing mice found that prenatal exposure may increase chances of those mice later developing pulmonary diseases (Wang et al., 2020). A study into the effects of exposure to both e-cigarette vapor and traditional cigarette smoke showed that mice exposed to both showed altered lung function, differing from even the effects of cigarette smoke alone (Lechasseur et al., 2020). An analysis of the relationship between e-cigarette exposure and cancer genesis has also been performed in mice. This study concluded that e-cigarette vapor potentially produced carcinogenic effects in the lung and bladder tissue of exposed mice, including lung adenocarcinomas (Tang et al., 2019).

#### Current Status on Flavor Induced Toxicology in Human *in vitro*

We could identify numerous studies wherein flavorant effects were observed after exposure to human cells in culture (Table 2). Cells treated with 10 μg/ml menthol e-liquid displayed a reduction in ATP levels in fibroblasts. A significant reduction in cell proliferation was observed between 24 and 96 h and cell migration at 72 h (Willershausen et al., 2014). A recent study measured menthol concentrations in all 68 e-liquids and showed a dose-dependent association with cytotoxicity (Al-Saleh et al., 2020). Lamb et al. (2020) reported menthol flavoring dependent mitochondrial dysfunction in BEAS-2B cells and successfully identified and measured the individual chemical constituents in the flavors through mass spectrometry that may be responsible for the effect. Menthol flavoring chemicals have been shown to cause endothelial cell dysfunction. In human aortic endothelial cells (HAECs) treated with the highest dose, 100 mmol/L of menthol resulted in a significant increase in cell death and IL-6 secretion. HAECs treated with concentrations of 0.001, 0.01, and 0.1 mmol/L menthol resulted in a significant decrease in nitric oxide production when stimulated with A23187, an endothelial nitric oxide synthase agonist. This indicates endothelial dysfunction since the increase in nitric oxide results in vasodilation and is an indication of cardiovascular health (Fetterman et al., 2018).

Menthol was previously known to inhibit the liver microsomal oxidation of nicotine to its metabolite cotinine, which can potentially lead to an increase in plasma levels of nicotine (MacDougall et al., 2003). In one study, an alveolar blood barrier consisting of a co-culture of epithelial lung cells on the apical compartment and endothelial cells on the basal compartment, was treated on the apical compartment with condensed e-cigarette aerosols of menthol and tobacco flavors with nicotine. Exposure for 24 h with the condensed aerosol identified as ‘Menthol 2’ resulted in a significant barrier dysfunction due to a reduction in transepithelial electrical resistance compared to both control and condensed aerosol base, composed of propylene glycol, vegetable glycerin, and nicotine. This reduction was not seen in the two tobacco condensed aerosols or the other menthol condensed aerosol, potentially indicating that interaction of menthol flavoring chemicals with other chemicals such as carvone (terpenoid) can increase cytotoxicity (Bengalli et al., 2017).

Neuronal stem cells (NSCs) treated with either e-liquids or aerosols in cell culture media of either tobacco or menthol flavors (1%) showed a significant increase in total autophagosome area compared to the control at both 4- and 24-h time points. Treatments with 0.5% menthol and tobacco e-liquids and menthol and tobacco six-total-puff equivalents increased percent lysosome co-localization. In turn, this potentially indicates a decrease in degradation and potential contribution to increased autophagic load (Zahedi et al., 2019). In HPdLF fibroblasts, exposure to Blu Classic Tobacco with 16 mg nicotine aerosol resulted in a significant increase in protein carbonylation while Blu Magnificent Menthol with 0 mg nicotine aerosol increased protein carbonylation but was not significant compared to the control. Meanwhile, IL-8 secretion and phosphorylated γH2A.X was increased in both Blu Classic Tobacco and Blu Magnificent Menthol aerosols (Sundar et al., 2016). Figures 1, 2 describe an overview of the mechanism of toxicity after menthol, mint and tobacco flavoring exposure.

In an additional study into the effects of exposure to general vapor from ENDS, damage to both lung epithelial cells and macrophages was noted. Following exposure, increased apoptosis and necrosis of the epithelial cells was observed, as well as increased cell death in the macrophages (Serpa et al., 2020).

Most studies reported reduced cell viability and/or increased pro-inflammatory mediators, although the study design did not include measurement of flavoring agent and therefore, more work may be needed to prove an association (Leigh et al., 2016; Yu et al., 2016; Leslie et al., 2017; Rowell et al., 2017; Behar et al., 2018; Otero et al., 2019; Go et al., 2020). Platelet-activating factor receptor (PAFR) mediated adhesion has been reported to increase in nicotine-free samples, although the study did not provide an experimental design assessing the tested tobacco flavor concentrations and exposure on the lung epithelial cell lines (Miyashita et al., 2018). No tobacco or menthol flavor associated effects were observed in human bronchial airway epithelial cells and human fetal lung fibroblasts (Lerner et al., 2015) and human monocytes (Muthumalage et al., 2017), though the concentration of flavoring agent in the aerosol were not measured. Flavors other than tobacco, mint, and menthol also displayed cytotoxicity in the tested cells. Cinnamaldehyde is the only chemical that consistently demonstrated cytotoxicity and increased cytokine release consistently (Table 2). We identified a few studies that demonstrated contrasting results and have tabulated them in Table 3.

## Challenges and Advantages Associated With E-Cigarette Flavor Ban

Despite pushing for the ban of flavored e-cigarettes and an FDA ban on the majority of flavors in e-cigarettes, concerns regarding e-cigarette users switching to combustion cigarettes has arisen. A longitudinal study looking at a group of adult e-cigarette users found that roughly 50% of participants reported that in light of the flavor ban they would attempt to “find a way to buy my flavor” or “add flavoring agents myself.” In addition, 9.6% of participants reported that “I would return to smoking traditional tobacco cigarettes” if there was a ban on all non-tobacco flavors (Du et al., 2020). In a discrete choice experiment using a population of adult smokers or recent quitters, it was observed that banning flavors in e-cigarettes while continuing to allow menthol in traditional cigarettes would result in an increase in 8.3% in traditional cigarette smoker, a decrease in 11.1% of e-cigarette use, and only 3% of participants would abandon both cigarettes and e-cigarettes (Buckell et al., 2018).

However, most young adults have reported that the first e-cigarette they used was flavored to taste like something other than tobacco. The most popular flavor reported was fruit and the second most common flavor reported was candy or dessert. In adults, tobacco-flavored e-cigarettes were more common compared to young adults and youth (Harrell et al., 2017). Despite the potential benefit of banning flavored e-cigarettes to reduce usage in youth and young adults, the usage may not decrease but rather a shift may be observed toward menthol or tobacco-flavored e-cigarettes.

It is also worth noting that regulating flavors of conventional cigarettes may also contribute to shifts in e-cigarette use. One online study found that among surveyed menthol cigarette smokers, approximately 15% reported that if a ban were placed on menthol cigarettes, they would most likely switch to using e-cigarettes (Wackowski et al., 2015).

Despite the challenges, studies have indicated that a governmental ban remains the most effective path to reducing the use of certain products or flavors. One study into an attempted “self-regulation” by one brand, where certain flavors were intentionally removed from the market, found that this strategy merely lead to consumers switching or other brands or increasing use of alternative flavors (Liber et al., 2020).

## Conclusion

Despite attempts by the United States government to curb the appeal of e-cigarettes to young people, the availability of menthol/mint-flavored e-cigarettes poses a potential issue. Menthol cigarettes are popular in adolescents as a result of reinforcement and thus nicotine dependence and e-cigarette use in youth and young adults continues. Despite the potential risk of adolescent use, a ban that would extend to menthol-flavored e-cigarettes would run a risk of pushing e-cigarette users back to traditional cigarette smoke. Future regulation of e-cigarettes needs to take into consideration the health effects of both tobacco and menthol flavors and any ban that would include menthol flavors with specific injurious chemicals would need to be in combination with a ban of menthol/mint cigars and cigarettes.

WHO document on assessment of menthol usage in tobacco products (including traditional cigarettes and e-cigarettes), published in 2018, emphasizes that restrictions should be imposed to other flavors in addition to menthol. A complete ban would reduce the potential shift to another flavor including menthol/mint. Although implementation of such a ban may vary dependent on a country’s economy, i.e., low-income, middle-income or high income. The ban on all tobacco products and flavoring additives will limit the likelihood that tobacco and menthol/mint use will simply shift to other products categories. However, flavor capsules (tobacco, menthol/mint and other flavors) are now gaining popularity due to lack of regulations. Canada, a high-income country, has implemented incremental restrictions on menthol usage in tobacco products and new product categories, i.e., ENDS have been clearly mentioned in the ban.

Tobacco, menthol, and mint have captured the ENDS market due to their aesthetic appeal, although guidelines for their use are largely lacking. Unfortunately, recent studies have reproducibly demonstrated cytotoxic effects in laboratory-based experimentation for these flavors. In addition, human reports studying the flavoring-based effects of ENDS products are yet to be conducted. We recommend that such human studies are required and should be conducted at the earliest opportunity to understand chronic exposure. In addition, earlier studies did not measure the flavoring agent concentrations or identify individual chemical constituents, possibly due to lack of proper detection methods. Newer studies are utilizing GC/MS based methods that may help in delineating any aerosol dose dependency based toxic effects. For example, flavors, especially cinnamaldehyde, are toxic in mice and *in vitro* human cell experiments, and warrant further investigation. Finally, we recommend that more studies are conducted with an experimental design based on the effect of individual flavor concentrations to make an accurate assessment.

## Author Contributions

GK and IR conceptualized the review. AG, TM, and MP prepared the figures and tables with the help from GK and IR. GK, AG, TL, MP, TM, and IR prepared the first draft of the manuscript. GK and IR finalized the manuscript. All authors contributed to the article and approved the submitted version.

## Conflict of Interest

The authors declare that the research was conducted in the absence of any commercial or financial relationships that could be construed as a potential conflict of interest.
