# Supplementary material for: Current Perspectives on Characteristics, Compositions, and Toxicological Effects of E-Cigarettes Containing Tobacco and Menthol/Mint Flavors
Source: Front Physiol. 2020 Nov 19;11:613948. doi: 10.3389/fphys.2020.613948 (PMC7710937; doi:10.3389/fphys.2020.613948)
Supplement: Supplementary file 1 [file Table_1.pdf]

Supplementary Table 1:

Flavoring agent containing ENDS. The table lists the current marketed e-liquids, e-liquids with salts, mods, pods and cartridges for tobacco, menthol and mint-based flavors. The product name and company are also listed.

Form	Flavor	Product name	Company/Brand/Store
E-liquid	Menthol	1. Dura Smoke Red Label<br>2. Dura Smoke Blue Label	Aleader X -DRIP(USA)
		3. Apollo Menthol Breeze E- liquid<br>4. Apollo Classic Tobacco Menthol E-liquid<br>5. Apollo Menthol E-liquid	Apollo E- cigs (USA)
		6. Absolute Zero<br>7. Arctic Blast<br>8. Fizzy Lemonade NS<br>9. Frost Tropic Freeze<br>10. Icy Mango NS<br>11. Lucy#7<br>12. Mountain Chill<br>13. Polar Plunge<br>14. Port Royal<br>15. Pure Ice	AVAIL VAPORS (USA)
		16. Farley's Gnarly Sauce Iced Out<br>17. Don't Care Bear Iced Out<br>18. The Lost One Cold Blooded	Bad Drip Labs (USA)
		19. Black Out City Blueberry Menthol Lemoncello	Bombies After Dark (USA)
		20. Candy King Ice Collection	Candy King Batch (USA)
		21. Diamond Mist E-Liquid 6 Mg Menthol<br>22. Iceberg Menthol	Diamond Mist (UK)
		23. Wizmix Menthol Asylum<br>24. Shortly Liqs Totally Menthol Shortfills<br>25. Wizmix Hut Green Slush Short<br>26. Wizmix Brain Freeze<br>27. Wizmix Berry Blizzard<br>28. Wizmix Black Currant Ice<br>29. Wizmix Cherry Chill<br>30. Wizmix Polar Bear<br>31. Button Junkie Coala Drool<br>32. Menthol Shortfill E Liquid Bundle<br>33. Blu E-Liquid Menthol<br>34. PTRNS ABYSS Shortfill	ECIGWIZARD (U.K)
		35. Menthol 18mg/Ml Nicotine E-Cig	E-burn (UK)
		36. Classic Menthol<br>37. Tobacco Menthol (Includes Both Blue And Red Label)	Dura smoke (USA)
		38. Alternativ Beta Liquid<br>39. Solace Black Purple Frost Nicotine Salt E-Liquid<br>40. Naked 100 Menthol Lovers E-Liquid Bundle<br>41. Vaporfi Mighty Menthol E-Liquid<br>42. Vaporfi Menthol Tobacco E-Liquid<br>43. Elementfrost E-Liquid<br>44. Vaporfi GRND RSRV Maui Menthol E-Liquid	Direct Vapor (USA)

		45. Joost Vapor House Line Menthol	
		46. Menthol 100% VG (E-Liquid Flavor)	Ecblend Flavors (USA)
		47. Ecovape Menthol "Strong" E-Liquid<br>48. Ecovape Menthol "Lite" E-Liquid	Ecopure (USA)
		49. Fin E-Liquid Menthol Ice<br>50. Fin Bold Menthol	Fin (Usa)
		51. Gamucci Premium E-Liquid Menthol (Regular, Smooth)	Gamucci (Uk)
		52. Cool Mist Menthol E-Liquid<br>53. Kringle's Curse E-Liquid<br>54. Menthol Ice E-Liquid<br>55. Mystic Menthol E-Liquid<br>56. Menthol V E-Liquid<br>57. Frost Bite Menthol E-Liquid<br>58. Krypton Menthol E-Liquid<br>59. Subzero - X Strength Menthol	Halo (Usa)
		60. Menthol<br>61. Menthol Chill<br>62. Menthol Clove	Hotvapes (USA)
		63. IQ Menthol	Ivape IQ (USA, U.K)
		64. Picnic Kalahari Ice Flavor Shot<br>65. Picnic Berry Mental E-Liquid Flavor Shot<br>66. Pure Menthol Flavor Shot<br>67. Picnic Heisen Bergerest E-Liquid Flavor Shot<br>68. Ice Menthol Jac Originals<br>69. Classic Menthol Jac Originals.	Jacvapour (U.K)
		70. J-Vapes Minthol<br>71. J-Vapes USA Menthol	J Vapes (USA)
		72. Cart CO Menthol<br>73. Docs Blend<br>74. Kik Menthol<br>75. Kik Menthol Sensation	Kik (U.K)
		76. Vaporx Menthol E-Juice	Krave (China)
		77. Cherry Menthol Flavor<br>78. Menthol Flavor	Lite Up Anywhere (U.K)
		79. Fumi Menthol By Fumizer<br>80. Dragonthol Salt By DNA Vapor<br>81. Blue Slushie Iced By Keep	Daddys Vapor (USA)
		97. Mad Vapor Hangsen Menthol<br>98. Mad Vapor, Mad Menthol<br>99. Prime Menthol	Madvape (USA).
		100. Mister E's Menthol	Mister E-Liquid (Usa)
		101. Menthol Additive<br>102. Twisted Menthol<br>103. Fruit Menthol<br>104. Menthol Ice	Mountain Oak Vapors (USA)
		105. Nic Quid Added Burst<br>106. Nic quid Bahama blizzard<br>107. Nic Quid mintoid<br>108. Nic Quid sinapse<br>109. Nic quid southern freeze.	Nicquid (USA)
		110. Maxi menthol<br>111. Turkish menthol<br>112. Wicked sick menthol	Nixteria (USA)
		113. Peeky blenders<br>114. Isickle e-liquid<br>115. Vgod e-liquid<br>116. Gangsta granny e-liquid	Simply e-liquid (U.K)

		117.ZOOM e-liquid hizenberg<br>118.Dr. Frost e-liquid<br>119.Lucky thirteen e-liquid<br>120.No frills e-liquid-Heisenberg	
		121.Arctic ice by mystic	Mystic (U.K)
		122.Mac menthol	River city vape (Canada)
		123.Menthol e-liquid	The vapor's knoll (USA)
		129. Menthol flavor e-liquid<br>130. Zero K menthol e-liquid<br>131. Mint to be menthol e-liquid	White cloud electronic cigarette (USA)
		132.Vapcigs menthol e-liquid	Vapcigs (USA)
		133.Blue menthol e-liquid<br>134.Dinner lady ice menthol e-liquid<br>135.Dinner lady cherry menthol salt nic e-liquid	Vape dinner lady (U.K)
		136.Vapecraft majestic menthol	Vapecraft (U.K)
		137.MTL blueberg<br>138.MTL menthol	Vapetek (Pakistan)
		139.Vapour shark menthol<br>140.Vapour shark extra menthol	Vapour shark (USA)
		141.Menthol Vapour2 e-liquid<br>142.Bold leaf menthol vapour 2 e-liquid	Vapour 2(U.K)
		143.Double Drip Menthol E-Liquid<br>144.Vapouriz Cool Menthol E-Liquid<br>145.Vapouriz Black Current & Liquorice Menthol E Liquid<br>146.Double Drip Menthol Mist Nic Salt E-Liquid<br>147.Vapouriz Double Menthol E-Liquid	Vapouriz (U.K)
		148.Puff E-Liquid Menthol	Puff E-Cig (USA)
		149.Menthol	Atmos (USA)
		150.Menthol	Ecto (USA)
		151.Black Menthol	Innevape (USA)
		152.Menthol	Flavor Artist (USA)
		153.Menthol	Upstate Vape (USA)
		154.Menthol	Aurelia (USA)
		155.Menthol	Exotic Vapors (USA)
		156.Menthol Tobacco	Kilo (USA)
		157.Menthol	Aelos (USA)
		158.Menthol	Next Smoke Juice (USA)
		159.Menthol	Aqua (USA)
		160.Xtreme Menthol	Next Vapor (USA)
		161.Vapourlites Menthol E-Liquid	Vapourlites (U.K)
	Tobacco	1. Apollo Tobacco Lovers<br>2. Apollo King Pin<br>3. Apollo Havana Nights<br>4. Apollo American Cigar<br>5. Apollo Simply Tobacco<br>6. Apollo RY4 E Liquids<br>7. Apollo Peanut Butter Tobacco<br>8. Apollo Classic Tobacco E Liquid<br>9. Apollo Triple Nickel E Liquid<br>10. Apollo Sahara E Liquid<br>11. Apollo Turkish Blend E Liquid<br>12. Apollo Vanilla Tobacco	Apollo E Cig (USA)
		13. Red Star Tobacco Classic<br>14. Gold Star Tobacco Classic<br>15. Orange Star Tobacco Classic<br>16. Blue Star Tobacco Classic<br>17. King Bellman	180 Smoke VAPE STORE (Canada)

		18. American Patriot<br>19. Tobacco Cuban Blend<br>20. Silver Star Tobacco Classic<br>21. Juno<br>22. Darts E-Juice<br>23. Va Bajo E-Liquid<br>24. Django E-Liquid<br>25. Grand Reserve E Juice Northern Classic	
		26. Port Royale	Avail Vapors (USA)
		27. Puff E-Liquid Tobacco	Puff E-Cig (USA)
		28. Hyde Tobacco E-Liquid	Hyde (USA)
		29. Beared Vape Co. Cappuccino Tobacco	Beard Vape (USA)
		30. CRFT Tobacco D'oro E-Liquid	CRFT (USA)
		31. Vaporfi Classic Tobacco<br>32. Vaporfi American Red Tobacco<br>33. Bold Tobacco E- Liquid By Tobacco Monster<br>34. Smooth Tobacco E-Liquid By Tobacco Monster<br>35. Rich Tobacco E-Liquid By Tobacco Monster<br>36. Tobacconist By Element Honey Roasted Tobacco E-Liquid<br>37. Tobacconist By Element Chocolate Tobacco E-Liquid<br>38. Tobacconist By Element Absinthe Tobacco E-Liquid<br>39. Brew's Brother E-Liquid By Motley<br>40. Euro Gold By Naked 100 E-Liquid<br>41. Caramel Tobacco By No Hype E Liquid<br>42. Yogi Vanilla Tobacco Granola E-Liquid<br>43. Cosmic Fog Chill'd Tobacco E Liquid	Direct Vapor (USA)
		44. Classic Tobacco<br>50. Dura Bacco<br>51. Havana Cigar<br>52. Renegade<br>53. RY4 Tobacco<br>54. Tobacco Cherry<br>55. Tobacco Cocoa<br>56. Tobacco Sweet<br>57. Tobacco Virginia (All Includes Both Blue And Red Label)	Dura Smoke (USA)
		58. Tobacco E-Liquid 18mg/Ml Nicotine E-Cig	E-Burn (UK)
		59. Wizmix Wizard's Leaf<br>60. Wizmix Popular Vape<br>61. Wizmix The Sherlock<br>62. Wizmix Havana Tobacco<br>63. Wizmix Dark Horse<br>64. Wizmix Pear Drops<br>65. Wizmix Simply Tobacco<br>66. Element Honey Roasted Tobacco<br>67. Element Hazelnut Tobacco<br>68. Wizmix Caramel Tobacco<br>69. Logic Pro Tobacco Capsules<br>70. Logic LQD Tobacco<br>71. Perfect Stranger Tobacco Twist Short Fills<br>72. Wizmix Nicotine Tester Pack<br>73. Blu E-Liquid Classic Tobacco	ECIGWIZARD (UK)
		74. Tobacco Blend 100%VG (E-Liquid Flavor)	Ecblend Flavor (USA)

		75. Leaf Red Tobacco (E -Liquid Flavor)<br>76. Leaf Tobacco Blend (E-Liquid Flavor)	
		77. Honey Roasted Tobacco E-Liquid By Element Ns10&Ns20<br>78. 555 Tobacco E-Liquid By Element Ns10& Ns 20	Element NS20 (U.K)
		79. E-Tron Tobacco E Liquid	E-TRON 3T (USA)
		107. Elo Tobacco<br>108. Kingside Tobacco<br>109. Royal Tobacco	Five Pawns (U.K)
		110.FIN Bold Tobacco<br>111.FIN USA Kentucky Tobacco<br>112.FIN British Gold Tobacco	FIN (USA)
		113.Firebrand Tobacco Royale<br>114.Firebrand Tobacco Gold	Firebrand (USA)
		115.Fum Tobacco	FUM (USA)
		116.Freedom Juice Tobacco E- Liquid<br>117.Prime 15 Tobacco E-Liquid<br>118.Torque 56 Tobacco E-Liquid<br>119.Tribeca Tobacco E-Liquid<br>120.Long Horn E-Liquid<br>121.Devlin E-Liquid<br>122.Pirate's Creed Tobacco E-Liquid<br>123.Voodoo Tobacco E-Liquid<br>124.Black Diamond E-Liquid<br>125.Timber Tobacco E-Liquid<br>126.Whisper Tobacco E-Liquid<br>127.Turkish Tobacco E-Liquid	Halo USA)
		128.55<br>129.Clove<br>130.Cowboy<br>131.Earth Blend<br>132.Gold Country<br>133.Illusion<br>134.Loco Tobacco<br>135.RY4<br>136.Tobacco Absolute	Hotvapes (USA)
		137.Iq Tobacco	Ivape Iq (Usa, U.K)
		138.Turkish Blend Tobacco Jac Originals<br>139. American Blend Tobacco Jac Originals<br>140. British Blend Tobacco Jac Originals	Jac Vapour (U.K)
		141.Bossman Russian Cream Tobacco	Juice Man (USA)
		142.Cured Tobacco<br>143.J Tobacco<br>144.Pursuit of Happiness<br>145.Pursuit of Madness<br>146.Pursuit of Sadness<br>147.Sweet Leaf Tobacco	J Vapes (USA)
		148.Kik Tobacco Blend<br>149.Kik Rolling Tobacco<br>150.Kik Smooth<br>151.Kik Virginian<br>152.Kik 88 Tobacco<br>153.Kik USA Red Tobacco<br>154.Kik Supreme Tobacco	Kik (U.K)
		155.Vaporx Tobacco E-Juice	Krave (China)
		156.Lite Up UK Blend Flavor<br>157.Lite Up UK Gold Flavor	Lite Up Anywhere (U.K)

		158. Lite Up UK Silver Flavor<br>159. Lite Up Virginia Blend Flavor<br>160. Lite Up French Pipe Flavor<br>161. Lite Up Toasted Blend Flavor<br>162. Lite Up Smooth Tobacco Flavor	
		163. Li-Queen Noble Tobacco	Li-Queen (U.K)
		164. Feared<br>165. Fumi Tobacco By Fumizer<br>166. Classic Tobacco Disposable E-Liquid By Twist<br>167. Pear Tobacco Disposable E-Liquid By Twist<br>168. The Cut LST SLT By Lost Art E-Liquid<br>169. Django By Spice Master<br>170. Apache Kid By Spice Master<br>171. Sacagawea By Spice Master<br>172. Geronimo By Spice Master<br>173. Jesse James By Spice Master<br>174. Belle Star By Spice Master<br>175. Butch Cassidy By Spice Master<br>176. 310 To Yuma By Spice Master<br>177. DOC Holiday By Spice Master<br>178. Billy The Kid By Spice Master	Daddys Vapor (USA)
		179. Prime Salts, Boujee Bacco<br>180. Prime Nickel Slots<br>181. Mad Vapor, Hangsen Congress<br>182. Panther Blend No-7<br>183. Alicious, Turkalicious<br>184. Alicious, Trubalicious<br>185. Mad Vapour, Sweet Carolina<br>186. Mad Vapour, Mad Tobacco<br>187. Mad Vapour Lucky 7<br>188. Madvapor Hangsen, RY4<br>189. QD VPR Classic Tobacco	Madvape (USA)
		190. Sweet Tobacco<br>191. Dry Tobacco<br>192. Classic Tobacco	Mi-Pod (USA)
		193. Casino 21<br>194. Copperhead<br>195. Elite<br>196. Grey Matter<br>197. HFC<br>198. Mr.-E's RY4<br>199. Pirates Bounty<br>200. Red Rider<br>201. Rough Neck<br>202. Southern Fields<br>203. Varadero	Mister E-Liquid (USA)
		204. Bourbon Tobacco By Salty Foc	Mountain Oak Vapors (USA)
		205. Nic Quid Mid Night Express<br>206. Nic Quid Gen-7<br>207. Nic Quid Daybreak<br>208. Nic Quid Soho	Nic Quid (USA)

209.Tradewinds Tobacco Cameroon E-Liquid<br>210.Tradewinds Tobacco Carolina E-Liquid<br>211.Tradewinds Tobacco Havana E-Liquid<br>212.Tradewinds Tobacco Scandinavian E-Liquid<br>213.Tradewinds Tobacco Turkish Tobacco E-Liquid<br>214.Drops Bold Tobacco E-Juice	Nicvape (USA)
215.555 Tobacco<br>216.About Midnight Tobacco<br>217.Burley Tobacco<br>218.Cuban Supreme Tobacco<br>219.Flue Cured Virginia Tobacco<br>220.Generic Tobacco<br>221.Maxi Tobacco<br>222.Mellow Sunset Tobacco<br>223.No-7 Tobacco<br>224.Perique Tobacco<br>225.Royal Tobacco<br>226.Tuscan Cigar Tobacco<br>227.Ry4 Tobacco	Nixteria (Usa)
228.POET Maple Spice Tobacco	P.O.E.T Electronic Nectar (USA)
229.Simply E-Liquid Tobacco<br>230.Simply E-Liquid Shortfill Tobacco	Simply E-Liquid (U.K)
231. Filipino Tobacco<br>232. Creamy Tobacco<br>233. Bold Tobacco<br>234. Peruvian Tobacco<br>235. Amalfi Tobacco<br>236. Washington Tobacco	Solace (USA)
237.American Red Tobacco E-Juice<br>238.Classic Tobacco E-Juice	South Beach Smoke (USA)
239.Analogue<br>240.Deadman<br>241.Resolution Salt Nic<br>242.Carmellow Tobacco<br>243.Nillabacco<br>244.Maplebacco<br>245.Pataspipe<br>246.Anarchy	River City Vape (Canada)
247.6th Street Tobacco USA E-Liquid<br>248.Cowboy Tobacco USA E-Liquid<br>249.Highway 555 USA E-Liquid<br>250.RY4 Tobacco USA E-Liquid	Texas Select Vapour (USA)
251.The Vapor Chef Tobacco E-Liquid	THE VAPOR CHEF (USA)
252.Regular Tobacco E-Liquid<br>253.Apache Tobacco E-Liquid<br>254.Atlantic Cut Tobacco E-Liquid<br>255.Bora Bora Tobacco E-Liquid<br>256.Cavendish Tobacco E-Liquid	White Cloud Electronic Cigarette (USA)
257.Wow Vapor Traditional Tobacco Juice	WOW Vapor (USA)
258.Yami Vapor-Kemuri	Yami Vapor (USA)
259.Vapcigs Tobacco E-Liquid	Vapcigs (USA)
260.Dinner Lady Cafe Tobacco E-Liquid	Vape Dinner Lady (U.K)
261.Vape Craft Rich Tobacco E-Liquid<br>262.Smokester Dark Tobacco Vape Juice	Vape Craft (U.K)

263.MTL Rollies<br>264.MTL Golden and Silver Cigs<br>265.Gold Rush<br>266.Sweet Tobacco<br>267.Butterbacco<br>268.Crumbacco.	Vapetek (Pakistan)
269.Vapour Shark Classic Tobacco<br>270.Vapour Shark Tobacco	Vapourshark (USA)
271.Red Tobacco Vapour 2 E-Liquid<br>272.Congress Tobacco Vapour 2 E-Liquid<br>273.Sahara Tobacco Vapour 2 E-Liquid<br>274.Gold Tobacco Vapour 2 E-Liquid<br>275.Black Tobacco Vapour 2 E-Liquid	Vapour 2 (U.K)
276.Double Drip Original Tobacco<br>277.Double Drip Caramel Tobacco<br>278.Vapouriz Silver Tobacco E-Liquid<br>279.Vapouriz Gold Tobacco E-Liquid<br>280.Vapouriz Rolling Tobacco E-Liquid<br>281.Vapouriz Original Tobacco E-Liquid<br>282.Vapouriz Virginia Tobacco E-Liquid<br>283.Vapouriz USA Gold Tobacco E-Liquid	Vapouriz (U.K)
284.Vapourlites Caramel, Cinnamon & Tobacco E-Liquid<br>285.Vapourlites Virginia Tobacco E-Liquid<br>286.Vapourlites Tobacco E-Liquid	Vapourlites (U.K)
287.VIP USA Kentucky Tobacco E-Liquid<br>288.VIP Virginia Hand Rolling Tobacco E-Liquid	VIP (U.K)
289.Vype Caramel Tobacco E-Liquid<br>290.Vype Smoky Leaf Tobacco E-Liquid<br>291.Vype Blended Tobacco E-Liquid	VYPE (U.K)
292.16 MG Tobacco<br>293.24 MG Tobacco<br>294.MG Tobacco	Encore (USA)
295.3&5<br>296.9X Tobacco<br>297.Texan Tobacco	Upstate Vape (USA)
298.American Tobacco	Ecto (USA)
299.Bold Cuban Tobacco	Innevape (USA)
300.Classic Tobacco	Next Smoke Juice (USA)
301.Classic Tobacco	ROC juice (USA)
302.Classic Tobacco	Vape dudes (USA)
303.Mild Tobacco	Next Vapor (USA)
304.Mild and Black	Exotic Vapors (USA)
305.Tobacco	Atmos (USA)
306.Tobacco	Vapor drops (USA)
307.Tobacco	Liquidbarn (USA)
308.Woodsy Blend<br>309.Robust & Strong<br>310.Turkish Blend	Royal Seven Tobacco (USA)
311.Tobacco	Aelos (USA)
312.Tobacco	Kalari – Vapor (USA)
313.Tobacco Lane Original Bold	Old Fashion Elixir (USA)
314.Tobacco Magic Flavor Enhancer<br>315.Flue Cured Virginia Tobacco<br>316.Tropical Tobacco<br>317.Rose Tobacco	EC Blend (USA)
318.True Blue<br>319.Smooth Tobacco	Kilo (USA)

		320. American Patriots<br>321. Cuban Blend	Tobacco Naked 100 (USA)
		322. Rose Tobacco	Aurelia (USA)
		323. Turkish Tobacco	Buck Naked (USA)
		324. Vanilla Tobacco<br>325. Coumarin Pipe Tobacco<br>326. East Coast Tobacco	Flavor West (USA)
		327. Sweet Tobacco	House E-liquids (USA)
	Mint	1. Cashman<br>2. Iced Coral<br>3. Green Freeze<br>4. Iced Mango Berries<br>5. Minty Apple Classic<br>6. Smooth Mint Classic<br>7. Heavy Menthol Classic<br>8. Slow Blow<br>9. Black Ice<br>10. Signature Collection Juno<br>11. Spring Mint<br>12. Brain Freeze<br>13. Very Cool<br>14. Polar Breeze<br>15. Fruit Medley Mint	180 Smoke Vape Store (Canada)
		16. SO GOOD Premium	Aleader X-Drip (USA)
		17. Fresh Farm E Liquid	Armageddon Mfg. (USA)
		18. DSRT Mint Chip Milkshake Max VG E Liquid<br>19. Smoozy Salt Perfectly Peachy Ice<br>20. Stryker OTM<br>21. Due Time Crisp Fuji Max VG E Liquid<br>22. Apollo Candy Cane E-Liquid<br>23. Apollo Melon Cucumber Mint E Liquid	Apollo E-Cigs (USA)
		24. The Mint Leaf	AVAIL VAPORS (USA)
		25. Pillow Mint (Smooth And Fresh Mint)	Bombies After Dark (USA)
		26. Bombies Mint	Bombies After Dark (USA)
		27. Salt Mint Iced Tea<br>28. Salt Mint	CRFT (USA)
		29. Diamond Mist E-Liquid Cool Mint	Diamond Mist (UK)
		30. Wizmix Spearminty<br>31. Blu E-Liquid Mint Chocolate<br>32. Blu E-Liquid Polar Mint	ECIGWIZARD (U.K)
		33. Fantasia E-Liquid Ice Mint	Fantasia (USA)
		34. FIN E-Liquid Bottle Subzero Mint	FIN(USA)
		35. Firebrand Peppermint Patty	Firebrand (USA)
		36. Spearmint E-Liquid	Halo (USA)
		37. Picnic Spearmint Bloo<br>38. Picnic Spearmint Flavor Shot	Jac Vapour (U.K)
		39. Kik Minty Bubblegum<br>40. Kik Ice Mint<br>41. Kik Spearmint<br>42. Kik Spearmint Short Fill<br>43. Kik Supermint	Kik (U.K)
		44. Apple Mint Flavor<br>45. Double Mint Flavor<br>46. Ice Mint Flavor<br>47. Spearmint Flavor	Lite Up Anywhere (U.K)
		48. Prime Pool Side<br>49. Salt Factory Mint	Madvape (USA)

		50. Madvapor, Ultra-Mint<br>51. Mad Vapor Spearmint	
		52. The Milkman Salt- Sweet Mint	Milkman (USA)
		53. Spearmint<br>54. Mightymint	Mi-Pod (USA)
		55. Bolly Sweet Mint	Mister E-Liquid (USA)
		56. Nicvape Heros Retro Spearmint	Nicvape (USA)
		57. After Dinner Mint<br>58. Cool Mint<br>59. Blueberry Mint<br>60. Winter Green Mint	Nixteria (USA)
		61. Mynto Ice By Drip Fire	Suorin Air (USA)
		62. Vapor For Life: Peppermint E -Juice By Wow Vapor	WOW Vapor (USA)
		63. Dinner Lady Spearmint E-Liquid	Vape Dinner Lady (U.K)
		64. Vapour Shark Cool Shot	Vapour Shark (USA)
		65. Peppermint Vapour 2 E-Liquid	Vapour 2 (U.K)
		66. Double Drip Spearmint E-Liquid<br>67. Vapouriz Ice Mint E-Liquid<br>68. Vapouriz Spearmint E-Liquid	Vapouriz (U.K)
		69. Vapourlites Ice Mint E-Liquid	Vapourlites (U.K)
		70. Cool Hit<br>71. Spearmint: 'Mint Selector'<br>72. Creme de Menthe	EC Blend (USA)
		73. Spearmint	Aurelia (USA)
		74. Misty Mint	Aelos (USA)
		75. Spearmint	Next Vapor (USA)
		76. Spearmint	Upstate Vape (USA)
		77. Spearmint	EVo (USA)
		78. Vype Crisp Mint E-Liquid.	VYPE (U.K)
E-liquid with salts	Menthol	1. Menthol Nic Salt E-Liquid By Dr Salt<br>2. Red Devil Nic Salt E-Liquid By Dr Salt	Dr Salt (UK)
		3. Menthol Ice By Mr. Salt	Mr. Salt (U.K)
		4. Yami Salt By Yami Vapor -Icy Trio	Yami Vapor (USA)
		5. SALT Glacier<br>6. Premium GOT Salt Candid Candy<br>7. Premium GOT Salt Ice Cap<br>8. Premium GOT Salt Purple Mojito<br>9. Premium GOT Salt POLAR	Vapetek (Pakistan)
		10. Dinner Lady Cherry Menthol Salt Nic E-Liquid	Vape Dinner Lady (U.K)
		11. Vapouriz Nic Salt Millionaire’s Menthol Nic Salt E-Liquid<br>12. Vapouriz Nic Salt Special Blend Nic Salt E-Liquid	Vapouriz (U.K)
		13. Subzero - X Strength Menthol	Halo Ultra Salts (USA)
	Tobacco	1. Salt Classic Tobacco	CRFT(USA)
		2. Gold & Silver Tobacco Nic Salt	Diamond Mist (UK)
		3. Smooth Tobacco Salt Nic E-Liquid by Tobacco Monster<br>4. Rich Tobacco Salt Nic E-Liquid by Tobacco Monster<br>5. Menthol Tobacco Salt Nic E-Liquid by Tobacco Monster<br>6. Amalfi Tobacco Nicotine Salt E-Liquid by Solace Salt<br>7. Filipino Tobacco Nicotine Salt E Liquid by	Direct Vapor (USA)

		Solace Salt<br>8. Peruvian Tobacco Nicotine Salt E-Liquid by Solace Salt<br>9. Washington Tobacco Nicotine Salt E-Liquid by Solace Salt<br>10. Creamy Tobacco Nicotine Salt E-Liquid by Solace Vapor<br>11. Apple Tobacco Salt by Pachamama Salt E Liquid<br>12. C&C Apothecary Signature Salted Caramel Tobacco E-Liquid<br>13. The Classic E-Liquid by Blu Salt of The Earth<br>14. American Patriots E-Liquid by Naked 100 Salt E-Liquid.	
		15. Bacco Nic Salt E-Liquid By Dr Salt	Dr Salt (UK)
		16. Smooth Nic Salt Pure Tobacco Flavors	Ecblend Flavor (USA)
		17. Ionic Salts<br>18. Riot Salt	Electric Tobacconist (U.K)
		19. Kwit Salt Nic Sweet Tobacco	Kwit Stick (USA)
		20. T Tobacco By Mr. Salt	Mr. Salt (U.K)
		21. Salt Nix Northern Tobacco	SALT NIX(Canada)
		22. Dinner Lady Smooth Tobacco Nic Salt E-Liquid<br>23. Dinner Lady Caramel Tobacco Nic Salt E-Liquid	Vape Dinner Lady (U.K)
		24. Vapouriz Nic Salt Virginia Tobacco E-Liquid<br>25. Vapouriz Nic Salt Original Tobacco E-Liquid<br>26. Vapouriz Nic Salt Classic Tobacco E-Liquid	Vapouriz (U.K)
		27. American Patriots<br>28. Tobacco	Naked 100 Salt e-liquids (USA)
		29. Tribeca - Smooth Tobacco<br>30. Turkish Tobacco - Robust Tobacco	Halo Ultra Salts (USA)
	Mint	1. I Love Salts Spearmint Gum Salt E-Liquid By Mad Hatter<br>2. Mint E-Liquid By Naked 100 Salts	Direct Vapor (USA)
		3. Mint By Mr. Salt	Mr. Salt (U.K)
		4. Kwit Salt Nic Mountain Mint	Kwit Stick (USA)
		5. Salt Nix Spring Mint	SALT NIX (Canada)
		6. Jewel Mint Diamond Salt By Pod Juice	Pod Juice (US)
		7. Mint	Salts by Keep it 100 (USA)
		8. Mint - Limited Edition	Air Factory Salts (USA)
		9. Spearmint	EVo (USA)
Mods	Menthol	1. AVS Star Bundle Menthol<br>2. Advanced Vaping Bold Menthol	FIN(USA)
	Tobacco	1. AVS Star Bundle Tobacco<br>2. Advanced Vaping Bold Tobacco	FIN(USA)
Pods	Menthol	1. Lushice Stig Disposable Pods Devices	AVAIL Vapors (USA)
		2. My Blu Tm Menthol Liquid Pod	Blu E-Cig (USA)
		3. My Blu Magnificent Menthol Liquid Pod	Blu E-Cig (USA)
		4. Menthol Pods	JUUL(USA)
		5. DOT Refill Pods -Menthol	Liberty Flights (U.K)
		6. NJOY Ace Pods: Menthol	NJOY (USA)
		7. Menthol Vapour Pods	Premium E-Cig (USA)
		8. Byte Pods Menthol	Totally Wicked (U.K)
		9. Menthol	Juno (USA)
		10. Menthol	Logic (USA)
		11. Menthol	Solo (VUSE) (USA)

		12. Menthol	Vuse (USA)
	Tobacco	1. LGND Grandpa's Choice<br>2. LGND Tobacco Row	AVAIL VAPORS (USA)
		3. My Blu Tm Tobacco Chill Intense Liquid Pod<br>4. My Blu Tm Tobacco Intense Liquid Pod<br>5. My Blu Tm Gold Leaf Liquid Pod<br>6. My Blu Tm Classic Tobacco Liquid Pod<br>7. My Blu Tm Carolina Liquid Pod<br>8. MyBlu Starter Kit – Tobacco<br>9. MyBlu Intense - Tobacco	Blu E-Cig (USA)
		10. Riptide Bright Leaf Tobacco Disposable Pods	Direct Vapor (USA)
		11. JUUL Golden Tobacco Pods	JUUL (U. K)
		12. Vype E-Pen 3 Pods Golden Tobacco	ECIGWIZARD (U.K)
		13. NS10 & NS 20 555 Tobacco Gusto Pod By Element.<br>14. NS10 & NS20 Honey Roasted Tobacco Gusto Pod By Element	Element NS20 (U.K)
		15. Virginia Tobacco<br>16. Classic Tobacco	JUUL (USA)
		17. DOT Refill Pods Tobacco	Liberty Flights (U.K)
		18. Sweet Tobacco Pods By Myle	MYLE (USA)
		19. NJOY Ace Pods: Classic Tobacco	NJOY (USA)
		20. Snowplus Classic Tobacco Pods	Snow Plus (U.K)
		21. Byte Pods -Tobacco	Totally Wicked (U.K)
		22. Tobacco	Cali Pods (USA)
		23. Tobacco	Juno (USA)
		24. Tobacco	Logic (USA)
		25. Rich Tobacco<br>26. Golden Tobacco	Vuse (USA)
		27. Original Blend	PHIX (USA)
	Mint	1. Stig Mighty Mint Disposable Pod Devices	STIG (USA)
		2. MYLE Iced Mint Pods	MYLE (USA)
		3. Snow Plus Classic Mint Pods<br>4. Snowplus Smokeless Pods	Snow Plus (U.K)
		5. Spearmint	Brewell MFG PHIX (USA)
		6. Mint	JUUL (USA)
		7. Mint	Sea Pods (USA)
Cartridges	Menthol	1. 21st Century Smoke Menthol 3ct-Cartridges<br>2. 21st Century Smoke Menthol 6ct-Cartridges	21st Century Smoke (Usa)
		3. Cigavette 5 & 50 Sealed Cartridges	Cigavette (USA)
		4. Green Smart Living Menthol E-Cig Vapor Cartridges	Green Smart Living (USA)
		5. Marvelous Menthol Prosmoke E-Cig Cartridges	Prosmoke (USA)
		6. Deluxe Cartridges Menthol	South Beach Smoke (USA)
		7. Menthol E-Cig Cartridges<br>8. Oasis Menthol E-Cig Cartridges<br>9. Virginia Menthol E-Cig Cartridges<br>10. Chillax Menthol E-Cig Cartridges<br>11. Congress Menthol E-Cig Cartridges<br>12. Dunwoody E-Cig Cartridges	Vapor For Life By WOW Vapor (USA)
	Tobacco	1. Cigavette 5 & 50 Sealed Cartridges	Cigavette (USA)
		2. Classic Tobacco Cartridges	Eversmoke (U.K)
		3. Green Smart Living Tobacco Gold E-Cig Vapor Cartridges<br>4. Green Smart Living Virginia Tobacco Cartridges	Green Smart Living (USA)

		5. Nicolites Refill Cartridges	Nicolites (U.K)
		6. Classic Tobacco Prosmoke Cartridges	Prosmoke (USA)
		7. Deluxe Tobacco Classic Flavor<br>8. Deluxe Tobacco Blue Flavor<br>9. Deluxe Golden Tobacco Flavor	South Beach Smoke (USA)
		10. STLTH Pod Cartridge-Tobacco Blend	River City Vape (Canada)
		11. VG Wow Boy E-Cig Cartridges<br>12. VG Traditional Tobacco E-Cig Cartridges<br>13. VG Triple Nickel Tobacco E-Cig Cartridges<br>14. Virginia E-Cig Cartridges<br>15. Richmond Tobacco E-Cig Cartridges<br>16. Hilltop E-Cig Cartridges<br>17. Dunwoody E-Cig Cartridges<br>18. Outlaw Blend E-Cig Cartridges<br>19. Traditional Tobacco E-Cig Cartridges<br>20. Congress E-Cig Cartridges<br>21. Oasis E-Cig Cartridges	Vapor For Life By WOW Vapor (USA)
	Mint	1. Vype E-Pen Cartridges	VYPE (U.K)
Cartomizer	Menthol	1. Cigirex E-Cigerette Cartomizer	Cigirex (USA)
		2. Ecopure Cartomizer	Ecopure (USA)
		3. Nicocig Refill Cartridges High Strength Cartomizers	Nicocig (U.K)
		4. Menthol Cartomizer	Premium E-Cig(USA)
		5. Menthol Cartomizer Single Pack	Vapcigs (USA)
		6. Hoxton Black Refills Menthol	Vapestick (U.K)
		7. Vapourlites Cartomizer Refill Pack Menthol	Vapourlites (U.K)
		8. VIP Menthol Refills Cartomizer<br>9. VIP Double Menthol Refills Cartomizer	VIP (U.K)
		10. 10motive Menthol Refills Cartomizer	Ecigwizard (U.K)
		11. Gamucci Micro 3 Cartomizer Refills Menthol	Gamucci (UK)
	Tobacco	1. Cigirex E- Cigerette Cartomizer	Cigirex (USA)
		2. Ecopure Cartomizer	Ecopure (USA)
		3. Nicocig Refill Cartridges High Strength, Medium Cartomizers	Nicocig (U.K)
		4. Premium Tobacco Cartomizer	Premium (USA)
		5. Tobacco Cartomizer Single Pack	Vapcigs (USA)
		6. Hoxton Black Tobacco Refills	Vapestick (U.K)
		7. Vapourlites Cartomizer	Vapourlites (U.K).
		8. VIP USA Tobacco<br>9. TEN MOTIVES USA Tobacco Refills	VIP (U.K)
Disposables	Menthol	1. Magnificent Menthol Disposable	Blu E-Cig (USA)
		2. Cigavette Menthol Flavored Disposables	Cigavette (USA)
		3. Gamucci Instant Use Menthol Disposable	Gamucci (UK)
		4. Xtra Disposable	Mi-Pod (USA)
		5. MYLE Slim Kit-Menthol Disposable Device	MYLE (USA)
		6. Menthol Premium Disposable	Premium E-Cig (USA)
		7. Prosmoke Menthol Flavored Disposable E-Cig	Prosmoke (USA)
		8. Dinner Lady Blue Menthol Disposable E-Cig	Vape Dinner Lady (U.K)
		9. Vapourlites VL5 Disposable E-Cigarette Menthol	Vapourlites (U.K)
		10. FLIQ- Disposable Vape Menthol.	Volcano E-Cig (U.K)
		11. Open Vape Repeat Subzero Menthol	Halovice (USA)
		12. Menthol	Fling (USA)
		13. Cool Menthol	NJOY (USA)
		14. Subzero Menthol	Vice (USA)
		15. Blue Razz	Zaero (USA)

		16. Menthol	
	Tobacco	1. Disposable Classic Tobacco	Blu (USA)
		2. Tobacco Series - American<br>3. Tobacco Series - Bold<br>4. Tobacco Series - Rich<br>5. Tobacco Series - Smooth	Hyde Color Edition (USA)
		6. Rich Tobacco	NJOY (USA)
		7. Tobacco	Puff Bar (USA)
		8. Old School Tobacco	Square XL (USA)
		9. Turkish Tobacco	The Jones (USA)
	Mint	1. Mint	Fresh.bar (USA)
		2. Icy Mint	Hype Bar (USA)
		3. Spear Mint	Hyde (USA)
		4. Cool Mint	Jolly (USA)
		5. Iced Mint	Phantom (USA)
		6. Spearmint	Sol (USA)
		7. Mighty Mint (Fresh Cool Mint)	STIG (USA)
		8. Minty Fresh	The Jones (USA)
Tanks	Menthol	1. Magnificent Menthol Blu Plus+Tank Tm	Blu E-Cig (USA)
	Tobacco	1. Gold Leaf Blu Plus+Tank Tm<br>2. Carolina Bold Blu Plus+Tank Tm<br>3. Classic Tobacco Blu Plus+Tank Tm	BLU E-Cig (USA)
Vaporizer	Mint	1. Barz Disposable Vaporizer	Yami Vapor (USA)

Recently, FDA issued warning letters to several companies who sell or distribute unauthorized ENDS products, which include XL Vape (Stig Inc), Flavour Warehouse Ltd (Vampire Vape), Pretty Women UK Ltd (T/A Coil2oil and Mad Kingdom), as well as disposable e-cigarettes: Puff bar, HQD Tech USA Lc, Myle Vape Inc (www.fda.gov).
